# The Tumor Microenvironment of DLBCL in the Computational Era

**DOI:** 10.3389/fonc.2020.00351

**Published:** 2020-03-31

**Authors:** Giuseppina Opinto, Maria Carmela Vegliante, Antonio Negri, Tetiana Skrypets, Giacomo Loseto, Stefano Aldo Pileri, Attilio Guarini, Sabino Ciavarella

**Affiliations:** ^1^Unit of Hematology and Cell Therapy, Laboratory of Hematological Diagnostics and Cell Characterization, Istituto Tumori “Giovanni Paolo II”—IRCCS, Bari, Italy; ^2^CHIMOMO Department, University of Modena and Reggio Emilia, Modena, Italy; ^3^Division of Haematopathology, European Institute of Oncology—IRCCS, Milan, Italy

**Keywords:** tumor microenvironment, transcriptomics, deconvolution, prognostication, DLBCL

## Abstract

Among classical exemplifications of tumor microenvironment (TME) in lymphoma pathogenesis, the “effacement model” resembled by diffuse large B cell lymphoma (DLBCL) implies strong cell autonomous survival and paucity of non-malignant elements. Nonetheless, the magnitude of TME exploration is increasing as novel technologies allow the high-resolution discrimination of cellular and extra-cellular determinants at the functional, more than morphological, level. Results from genomic-scale studies and recent clinical trials revitalized the interest in this field, prompting the use of new tools to dissect DLBCL composition and reveal novel prognostic association. Here we revisited major controversies related to TME in DLBCL, focusing on the use of bioinformatics to mine transcriptomic data and provide new insights to be translated into the clinical setting.

## Introduction

Diffuse large B cell lymphoma (DLBCL) has long been regarded as a paradigm of aggressive diseases composed of malignant B cells dividing rapidly and independently of stimuli from the surrounding tumor microenvironment (TME) (1). Over the last few years, evolving technologies enabling deeper genomic and transcriptomic profiling revealed an underestimated complexity of DLBCL biology, involving both the malignant and non-malignant compartments of the disease.

Seminal gene expression profiling (GEP) studies showed striking associations between expression of genes reflecting tumor cell-of-origin (COO) and outcomes to standard immuno-chemotherapy (2). Two distinct molecular categories, germinal center B cell and activated B cell (ABC), were incorporated in the revised WHO classification of DLBCL (2). The development of immunohistochemistry (IHC) algorithms to surrogate GEP was promptly followed by the commercialization of a gene panel for proper COO determination (3). The assessment of the transcriptional, rather than phenotypical, features of DLBCL also resulted in a remarkable improvement of survival prediction. With the advent of new technologies, such as NanoString, the digital gene expression measurement on formalin-fixed paraffin-embedded (FFPE) biopsies facilitated the inclusion of COO categorization in daily clinical practice (3). However, very recent integrative analyses by whole-exome and transcriptome sequencing brought DLBCL genetics to a new level (4–6), identifying molecular categories within COO classification characterized by distinct drivers with novel prognostic and therapeutic implications.

While a substantial amount of information from these studies is being translated into the clinic, results capturing the molecular aspects of TME are still under debate. Historical GEP analyses provided alternative categorization of DLBCL based on the differential expression of genes reflecting inflammatory host response and oxidative metabolism (7) or enrichment in peculiar immune and extra-cellular determinants (8). Such observation remained poorly applied on clinical ground owing to an incomplete comprehension of the specific cellular/molecular TME determinants and the precise mechanisms of their prognostic impact. In a recent translational effort, our group exploited a computational approach to reinterpret large transcriptional data and provide a pure TME-based prognosticator that improves the COO risk stratification (9). Latest results from sequencing studies and clinical trials on new drugs (i.e., lenalidomide and ibrutinib) underscored the relevance of studying DLBCL heterogeneity, taking into proper account the impact of TME in diagnostics, prognostics, and therapeutic prediction.

We reviewed current controversies related to TME in DLBCL, with particular emphasis on recent computational strategies capturing new microenvironmental features, at both the cellular and the molecular levels.

## Evolving Technologies to Face TME-Related Controversies

About 20 years ago, the first GEP-scale analysis of *de novo* nodal DLBCL not otherwise specified demonstrated that morphological approaches, even supplemented by IHC, were incapable of capturing divergent molecular modules between tumors, and identified two main subgroups resembling the diverse stage of B cell differentiation with a different prognosis (10). Each subgroup also showed consistent transcriptomic heterogeneity of non-malignant compartment. The expression level of many genes reflected a variable extent of T cell (*TCR-beta, CD3e, Fyn, LAT, PKC-u*), monocyte/macrophages (*CD14, CD105, CSF-1R, FcR-gamma*), and natural killer (*NK4*) infiltration as well as extracellular matrix (ECM) remodeling by metalloproteinases (i.e., *MMP9* and *TIMP*), integrins, chemokines, and other stromal axes (i.e., CXCR4/SDF-1). Assembled in the so-called lymph node (LN) signature, these genes were shared by samples of normal lymph nodes and tonsil (10), remarking their structural and immune function within secondary lymphoid organs (SLO) (11). A second large genome-scale study highlighted a direct correlation between the expression of the LN signature and a better outcome after CHOP chemotherapy, emphasizing that the ABC subgroup had the lowest enrichment of genes in the signature (12). Their expression was also inversely related to a “proliferation” signature including genes regulating malignant growth processes and *BMP-6*, a single TGF-related mesenchymal gene associated with poor outcome. Once again, stromal factors implicated in ECM organization and shared by elements of innate immunity, especially macrophages (Mo), dendritic cells (DC), and NK, were involved in the physiopathology and drug response of DLBCL. This observation was partially confirmed by Monti et al. (7) who identified a “host response” gene set in DLBCL, showing a coordinated activation of inflammatory response driven by CD3^+^ T cells, DC, Mo, and NK, adhesion axes (LFA-1, PECAM-1, and SDF-1), cytokine/chemokine stimuli (especially IFN and TNFα), and ECM components (i.e., collagens). However, the patients in this cluster did not show any therapeutic advantage following CHOP chemotherapy. A subsequent work by Lenz et al. (8) definitely recognized a “stromal” signature related to the sorted CD19-negative non-malignant component, reflecting high deposition of ECM proteins, as fibronectin (FN), secrete protein acid rich in cysteine (SPARC), and various collagen isoforms and prevalent infiltration of cells of myelo-monocytic lineages. DLBCL expressing this signature showed longer survival after R-CHOP independently of COO, suggesting an intriguing stromal protection and raising the question on whether the abundance of histiocytes prompts the tumor cell killing by rituximab.

Beyond their relevance in characterizing the tumor cell fraction, GEP results strengthened the idea that finely regulated interconnections between mesenchymal (stromal) and hematopoietic (immune) counterparts in SLO govern the extent of inflammatory reactions as the tumor evolves. Such underestimated mechanisms were likely independent of COO and seemed to underlie the inter-patient diversity in drug responsiveness. Measuring selected TME genes by RNA microarrays, however, remained mechanistically uninformative and, although of certain prognostic utility, was hampered by cost, standardization issues, and scarce availability of fresh–frozen biopsy material. Great translational efforts, in fact, were devoted at surrogating GEP by flow cytometry or IHC and localizing cellular contributors of TME-based prognostication directly on FFPE material. *In situ* staining of matricellular proteins, such as FN, SPARC, and collagens, as well as IHC or immunofluorescence (IF) quantification of tumor-infiltrating lymphocytes and other immune cells (13–17) provided results partially in line with GEP, but highly controversial due to their low reproducibility and questionable validation. They further underscored that the static pictures of protein or surface marker expression are inadequately representative of the transcriptional dynamism that controls TME components at functional rather than phenotypic level. This aspect is particularly critical for Mo and explains their controversial role in DLBCL prognostication (18). When measured by the sole CD68 IHC staining, the extent of tumor infiltration by Mo appeared significantly associated with an adverse outcome to CHOP therapy only in the study by Cai et al. (19), whereas it had no prognostic value in other studies (13, 20, 21). Conversely, CD68 at both the RNA and the protein levels was found to have a positive prognostic impact in patients treated by rituximab plus CHOP (22). Co-staining of CD68 and CD163—capturing putative immunosuppressive Mo with a M2-like phenotype—correlated with shorter survival in R-CHOP-treated cohorts (23–25), whereas the prevalence of either M1-like CD68^+^/HLA-DR^+^Mo (24) or M2-like CD163^+^ cells in similar studies did not show any significant prognostic association (22). Such discrepancies not only were mainly due to differences in staining techniques, antibody clones, patient cohorts, and treatments, but also imply that simple detection of surface molecules does not surrogate the extreme *in vivo* functional plasticity of Mo. Recently, a “lymphoma-associated Mo interaction gene” signature (LAMIS) was built on pooled GEP datasets and associated to shorter PFS and OS in a large cohort of R-CHOP/R-CHOP-like-treated patients, independently of COO and IPI status (26). However, beyond prognostic implications, a fundamental comprehension of Mo biology is still lacking, probably due to insufficient technology to disentangle their quantitative, functional, and phenotypic dynamics within the DLBCL milieu.

On the other hand, as the access to huge amounts of transcriptomic data from bulk tissues became available, the application of new computational tools allowed unprecedented degrees of TME exploration. The deconvolution of GEP or RNA sequencing (RNA-seq) data was shown to provide simultaneous information about quantitative proportions of non-malignant cell types and their transcriptional states, uncovering potential prognostic and therapeutic associations (27–29). In a direct experience of our group, publicly available GEP datasets, including the one by Lenz et al. (8), were analyzed by CIBERSORT (27) to draw maps of the immune/stromal ecosystem in more than 480 R-CHOP-treated DLBCL. Then, the identification of prognostic genes—associated to commonalities between cases in estimated fractions of specific microenvironment cytotypes—represented the first approach exploiting deconvolution to overcome the limits of GEP. Moreover, the prognostic power of the panel was validated by NanoString technology on two independent patient cohorts and demonstrated the feasibility of measuring the expression of TME-related transcripts directly on FFPE diagnostic biopsies (8). An innovative deconvolution framework using CIBERSORTx (29) to combinations of single-cell RNA-seq and bulk transcriptomic data has been very recently reported in *de novo* DLBCL. This approach recognized 49 distinct transcriptional states across 13 main tumor-associated cytotypes, including neutrophils, Mo, fibroblasts, and T cells (30). Patient subsets with peculiar enrichment in TME cell states also showed significant outcome differences that cannot be identified by classical transcriptomics. Consistently, the preliminary results from an independent investigation—applying an alternative algorithm to deconvolve >3,000 DLBCL from 13 transcriptomic and mutational datasets—identified four lymphoma subclasses with distinctive TME traits pairing recurrent genetic drivers of the tumor. Moreover, these new categories show different outcomes, independently of recent molecular classification (31). Such pioneering methods to unify subtle changes in rare TME populations with genetic features of the malignant counterpart provide unprecedented insights in DLBCL biology but require additional effort to prompt their clinical and even therapeutic applicability. Table 1 summarizes the major published studies exploring DLBCL TME over the last 20 years.

**Table 1:** List of studies assessing the prognostic implication of TME in DLBCL.

**Technique**	**Biomarker**	**TME component**	**Number of cases/material**	**Treatment**	**Prognostic implication**	**References**
IHC/IF	CD1a+ (DC) Granzyme B^+^ (T cells)	DC	48/FFPE	CHOP/Rituximab	CD1a+: favorable OS Granzyme B+: favorable OS	(23)
	SPARC CD68	Stromal cells	262/FFPE	R-CHOP/R-CHOP-like	SPARC: favorable OS and FS CD68: not significant	(13)
	SPARC FN1	Stromal cells	173/FFPE	CHOP/CHOP-like R-CHOP/VACOP	SPARC/FN1: favorable OS	(14)
	FOXP3 CD3	T cells	161/FFPE	R-CHOP	FOXP3 and CD3: favorable	(21)
	PD-1 CD3 PD-L1	T cells	414/FFPE	R-CHOP	CD3^high^PD-1+: unfavorable OS PD-1/PD-L1 interaction: unfavorable OS	(32)
	PD-1 LAG-3 TIM-3	T cells	123/FFPE	R-CHOP/other	TIM-3: unfavorable OS and PFS	(33)
	CD68 CD163	Mo	221/FFPE	CHOP/R-CHOP	CD68: unfavorable OS and PFS CD163: unfavorable OS and PFS	(34)
IHC GEP	CD68 CD163	Mo	181/FFPE (IHC) 544/FF (GEP)	R-chemo Chemo	R-chemo: favorable PFS and OS Chemo: unfavorable PFS and OS	(22)
GEP	Lymph node T cell signatures	Monocyte/Mo NK ECM T cells	42/FF	Anthracycline-based regimens	-	(10)
	Host response signature	T cells, monocyte/Mo, DC	176/FF	CHOP	Unfavorable	(7)
	Stromal-1 Stromal-2 signatures	ECM proteins Mo Vascular density	414/FF	CHOP/R-CHOP	Stromal-1: favorable Stromal-2: unfavorable	(8)
	LAMIS signature	Mo	466/FFPE	R-CHOP/R-CHOP like	Unfavorable	(26)
RNA-seq IHC	PD-L1	Mo	702/FFPE (RNAseq) 433/FFPE (IHC)	R-CHOP vs. obinutuzumab-CHOP and R-CHOP±bevacizumab	Favorable	(35)
Deconvolution (CIBERSORT)	45-TME gene panel	Myofibroblasts DC CD4-T cells	482/FF 215/FFPE	R-CHOP/R-CHOP like	Favorable OS and PFS	(9)

## Biological Determinants of TME-Related Prognostication

Taken together, results from both low- and high-resolution dissection of DLBCL outlined aspects of TME dynamics that remained underestimated for years. Molecular signatures reflecting a predominant fibroblastic reaction and Mo infiltration correlated with better outcomes, thus generating a paradoxical interpretation of the common meaning of tumor-associated fibroblasts and Mo (36). Our recent work also emphasizes that biological differences between cases in the validation cohorts may impact on prognosis since they were homogeneously selected based on molecular and clinical parameters [i.e., the validation sets include only advanced-stage patients who have undergone standard front-line R-CHOP/R-CHOP-like regimens (9)].

From a biological point of view, ECM components as well as fibroblasts and Mo appear critically inter-chained as major cross-players of the structural and inflammatory machineries of SLO. Collagens, proteoglycans, glycoproteins, metalloproteinases, and matricellular proteins, such as SPARC and osteopontin, are synthesized by mesenchymal elements and partially by Mo, generating heterogeneous mixtures undergoing continuous remodeling under the pressure of tumor growth and inflammation (37). The deposition of non-cellular factors also mediates the activation of adhesion molecules and integrins (i.e., αVβ3 or α6β4) that provide anchorage to Mo and T cells and possible antigen-independent stimulation of the BCR pathway in malignant cells (38). Paracrine gradients of cytokines and chemokines released by stromal and tumor cells themselves also drive the recruitment and the polarization of monocytes/Mo, T cells, DC, as well as other stromal elements with antigen-presenting capacity, such as follicular dendritic cells and fibroblastic reticular cells (FRC) (39–41). A number of preclinical studies indicated that accessory cells as neutrophils, stromal cells, monocytes, and T cells hold the capacity to modulate tumor survival. Neutrophils can be recruited by CXCL8-secreting tumor cells and, in turn, modulate tumor growth by secreting the proliferation-inducing ligand APRIL and up-regulating the NF-kB, STAT3, and p38 pathways via the Toll-like receptor 9 signal (42–44). Co-cultures of mouse stromal elements with primary DLBCL cells enhanced their clonogenicity as effect of both cell-to-cell adhesion and paracrine mechanisms involving the B cell activating factor and the BCL2 axes (45, 46). Similarly, cells of monocytic origin were proved to prolong lymphoma cell survival by mechanisms that are still unclear (47). All these models, however, remain poorly representative of the *in vivo* complexity of tumor/TME interactions and far from explaining their influence on outcome to standard immunochemotherapy.

An additional influence of TME on lymphoma behavior involves the defective immune competence of effector cells. A PD-L1 overexpression by tumor and TME components is observable in a considerable fraction of DLBCL showing pools of exhausted PD-1^+^ T cells (48). The phagocytic activity of Mo and DC is likewise hampered by SIRPα stimulation after binding with CD47, which is up-regulated on tumor cells. Both these mechanisms encouraged the experimental use of new anti-PD-1 and anti-CD47 antibodies in relapsed/refractory DLBCL, aiming at restoring the specific immune function of TME (49, 50).

On the other hand, some *in vitro* and *in vivo* results suggest the ability of tumor cells to shape the composition of the surrounding milieu. For instance, genetically unstable DLBCL cells display reduced surface expression of MHC and CD58 molecules, thus lowering T cell and NK infiltration and cytotoxicity (51). Conversely, DLBCL-released lymphotoxins and TNF-alpha were reported to promote the proliferative attitude of podoplanin-, PD-L1/L2-positive fibroblasts, while lowering their ability to contract collagen fibers and attract cytotoxic T cells (52).

**Figure 1:**
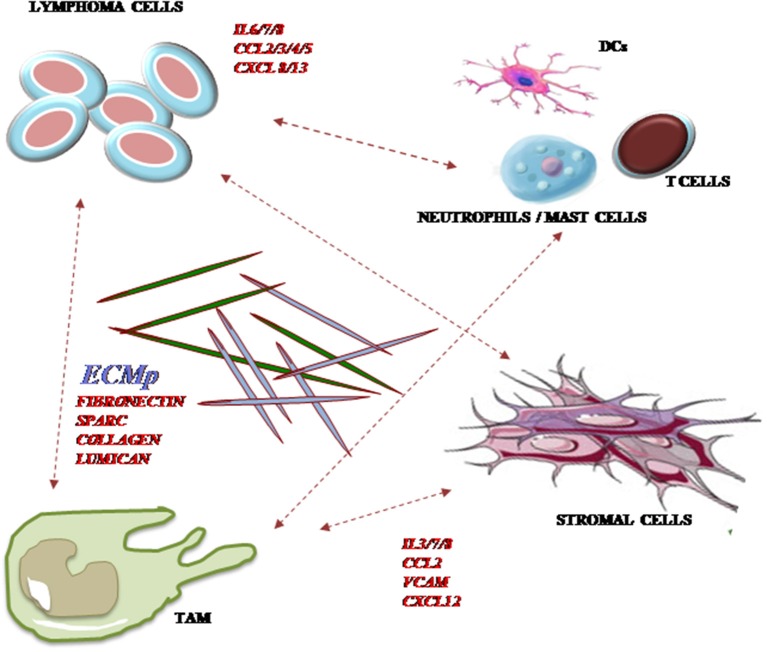
Major cellular and non-cellular components of TME in DLBCL. A number of cellular elements of both immune and stromal origins generate intricate cell-to-cell and paracrine networks with tumor B cells. Reciprocal modulation occurs between malignant clones and tumor-associated macrophages (TAM), stromal and immune cells including neutrophils, mast cells, T cells, and dendritic cells (DC) through the expression of chemokines, cytokines, and extracellular matrix (ECM) component deposition. CCL, CC-motif chemokine ligand; CXCL, CXC-motif chemokine ligand; CXCR, CXC-motif chemokine receptor; ECM, extracellular matrix; IL, interleukin.

Overall, it is conceivable that the local extent of constitutional and reactive processes of both stromal and inflammatory nature shapes the final cellular composition of the affected lymph nodes, forming specialized contextures with topographical and functional identity (Figure 1). These niches may vary within the same tumor, across different tumor sites in the same patients, and between different patients, resulting in a relevant biological and outcome diversity. The application of innovative computational tools (9, 26) added texture to this picture in DLBCL, yet remaining elusive about the precise mechanisms and timing of TME-centered dynamics. The recognition of a single biological trait unifying the complexity of tumor/TME interactions is very challenging, owing to their potential variation at different disease stages and type of treatment. In fact, the favorable prognostic value observed for stromal/Mo signatures in DLBCL treated by chemo-immunotherapy may rely on the mechanism of rituximab action, which activates killing by the phagocytic capacity of resident immune cells, especially Mo (53). There is indeed growing interest in exploring the role of pure stromal axes, such as SDF-1/CXCR4, in sustaining B cell survival via BCR-independent mechanisms (54) and affecting their sensitiveness to BCR inhibitors (i.e., ibrutinib) and immune modulators (i.e., lenalidomide) with a known off-target effect on both the stromal and the immune components of TME (55, 56).

## Conclusion and Future Directions

Enormous body of work based on new-generation technologies has produced low/medium-resolution data on the quality of tumor and its surrounding TME, to predict patient responsiveness to standard therapy. While the success of novel immunotherapies increases in other lymphoma subtypes, clinical results are unsatisfactory in DLBCL. Therefore, characterization of TME is emerging as a critical step for strengthening the rationales of upcoming treatments or enriching subgroups of front-line responder patients. The implementation of computational techniques offers a chance to mine old bulk transcriptomic data and interrogate new sequencing records at a single-cell level. Moreover, the combination of innovative multidimensional applications of digital pathology is expected to provide deeper insights on the composition, function, and localization of immune and stromal determinants of DLBCL.

On the other hand, despite tremendous experimental efforts, it remains of critical importance to clarify (i) whether and how the tumor transcriptional, mutational, and immunogenic landscape influences the TME composition; (ii) how reciprocal stimuli between tumor and immune/stromal cells change as the disease progresses and under the influence of different drugs; and (iii) how the constitutive local feature of the SLO microenvironment influences tumor initiation and progression. Robust preclinical models and *in vivo* ultra-sensitive arrays to measure subtle TME changes will be necessary to answer these questions and translate future results to the clinical setting.

## Author Contributions

SC and GO discussed, designed, and conceptualized the review article. SC, GO, and MV wrote and edited the article. AG provided the first revision of the article. Literature review was performed by TS, GL, and AN. AN and TS respectively constructed Figure 1 and Table 1. SC and SP performed the final revision of the article.

### Conflict of Interest

The authors declare that the research was conducted in the absence of any commercial or financial relationships that could be construed as a potential conflict of interest.
